# B-cell centrality dictates therapeutic efficacy across autoimmune diseases: a systematic review

**DOI:** 10.3389/fimmu.2026.1906254

**Published:** 2026-09-10

**Authors:** Vishakha Hooda, Harsh Goel, Taruna Madan, Alpana Sharma

**Affiliations:** 1 Department of Medical Oncology, All India Institute of Medical Sciences, New Delhi, India; 2 Laboratory Oncology Unit, Dr. B.R. Ambedkar Institute Rotary Cancer Hospital, All India Institute of Medical Sciences, New Delhi, India; 3 Division of Development Research, Indian Council of Medical Research, New Delhi, India

**Keywords:** autoimmunity, B cell therapy, BTK inhibition, CAR T, combinatorial therapy, myasthenia gravis, pemphigus, rheumatoid arthritis

## Abstract

**Background:**

B cells play a critical role in autoimmunity through autoantibody production, plasma cell differentiation, antigen presentation, cytokine secretion, and germinal center responses. The clinical efficacy, durability, and safety profiles vary across autoimmune diseases. Therefore, the objective is to assess B-cell therapeutic responses and their correlation with disease pathology in autoimmunity.

**Methods:**

Embase, Web of Science, and PubMed were systematically screened for clinical trials published between 2020 and July 2025. A total of 24 clinical trials across five autoimmune conditions—pemphigus vulgaris, myasthenia gravis, rheumatoid arthritis, systemic sclerosis, and Sjögren’s syndrome—were evaluated for B-cell-targeted therapy. Interventions included B-cell depletion therapy, CAR-T cell therapy, BTK inhibition, rituximab and its biosimilars, and combination strategies.

**Results:**

Across 24 clinical trials, more than 3,500 participants were included. We observed that autoantibody-mediated diseases pemphigus vulgaris and myasthenia gravis had the most superior and durable immune response to B-cell depletion by rituximab and inebilizumab, respectively. BTK inhibition showed rapid but non-durable responses. Immune complex diseases like rheumatoid arthritis and systemic sclerosis showed improvement in activity scores and partial response, and no clinical remission after treatment with rituximab and its biosimilars. The next-generation approach BCMA-directed CAR T-cell therapy showed promising results with potential durability in myasthenia gravis. Combination therapy using belimumab and rituximab resulted in a significantly better clinical outcome than monotherapy in Sjögren’s syndrome. Overall, B-cell therapy safety profiles showed no severe adversity and were well tolerated.

**Conclusions:**

The success of B-cell therapeutics appears to align with B-cell contribution, disease biology, and the depth of B-cell targeting in autoimmune conditions. Precision targeting is needed to effectively combat autoimmune diseases.

## Introduction

B cells or B lymphocytes are important immune cell subsets around which the whole immune system revolves. From producing antibodies against pathogens to secreting key cytokines, not only do they help other immune cells in their optimal functioning, but they also serve as antigen-presenting cells to T cells (1). B cells are produced in the bone marrow wherein they develop variable immunoglobulin receptors to target diverse groups of pathogens by neutralization, opsonization, antibody-mediated and cell-mediated cytotoxicity, or promoting phagocytosis (2, 3). During this process, self-reactive B cells are either suppressed or eliminated to develop tolerance against self-antigens. However, like many biological processes, the tolerance against autoreactive B cells is not infallible, leading to autoimmunity and other malignancies. Autoreactive B cells, therefore, directly—by producing autoantibodies—or indirectly—by secreting cytokines and engaging with T cells as antigen-presenting cells—produce abnormal immune response contributing to autoimmune pathology (4). Therefore, in the 1900s, B-cell depletion was adopted as one of the approaches to target autoimmunity (5).

**Figure 1:**
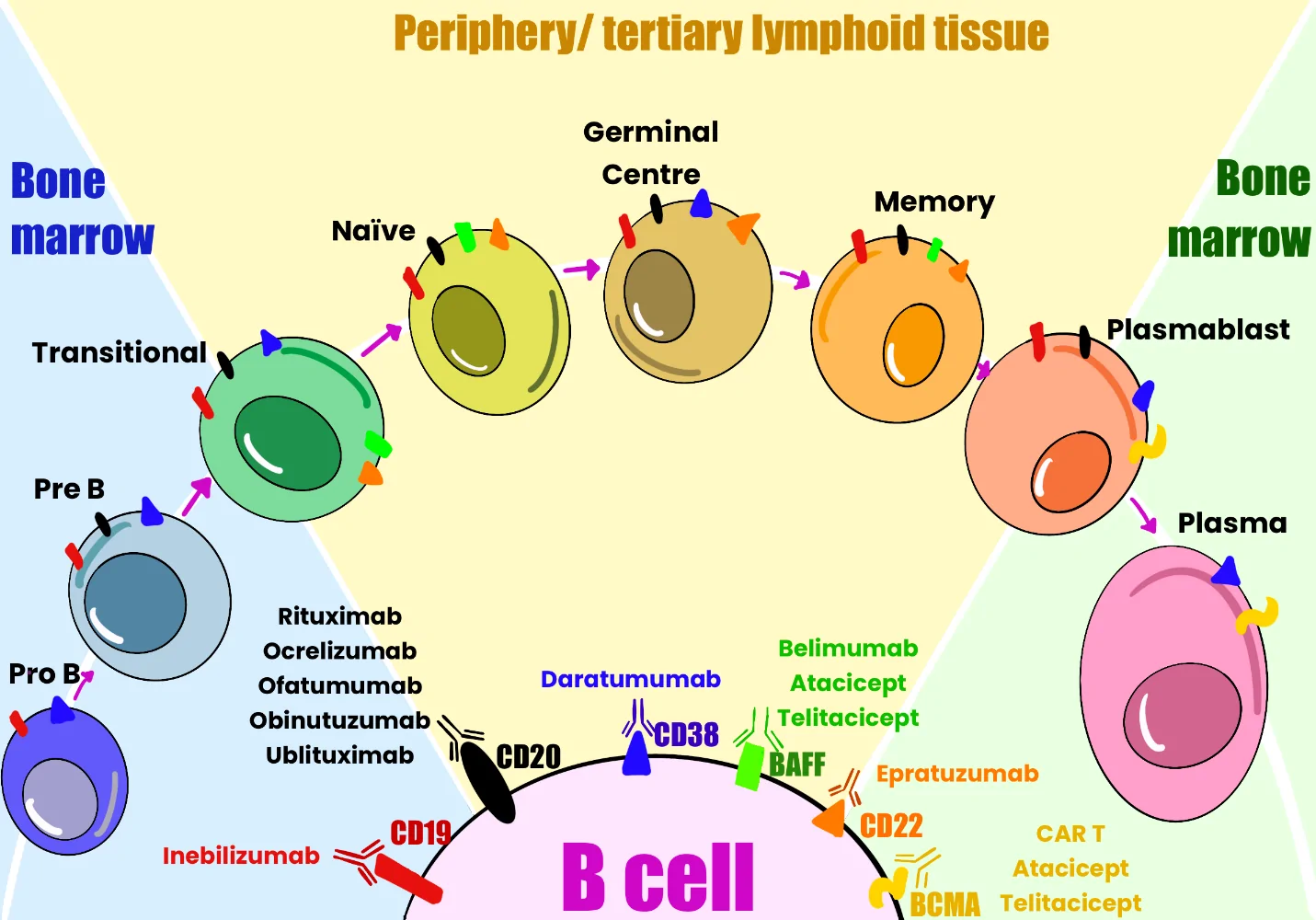
B-cell developmental stages and corresponding therapeutic targets. Image depicting schematic representation of B-cell stages from progenitor B cell (Pro B), precursor B cell (Pre B), transitional, naïve, germinal center, memory, plasmablast, and plasma cells along with common B-cell therapeutic targets like CD19, CD20, CD38, CD22, BAFF, and BCMA and their corresponding B-cell therapy.

Owing to disease heterogeneity and the patient’s variability to treatment response, B-cell depletion therapy is of many types. B-cell-targeted therapeutics involves direct depletion of B cells using monoclonal antibodies such as anti-CD20 [rituximab (RTX)] and indirect depletion by targeting activation markers, cytokines, or co-stimulatory markers to dampen B-cell responses (**Figure 1**) (6–9).

Direct depletion by mAb may include RTX, ocrelizumab, ofatumumab, and ublituximab, which targets CD20 on B cells (10). In rheumatoid arthritis (RA), RTX is approved for patients whose disease remains active despite TNF inhibitor therapy, where it has been shown to deliver long-term control of inflammation and joint symptoms (**Table 1**) (25, 26). In pemphigus vulgaris (PV), CD20 blockade has become a standard first-line therapy for moderate-to-severe cases, frequently leading to sustained remission (27, 28). Studies in primary Sjögren’s syndrome (pSS) have yielded mixed findings, though some have reported symptom relief and partial restoration of glandular function (29, 30). In systemic sclerosis (SSc), RTX treatment has been linked to a measurable reduction in skin thickening and preservation of lung capacity (31, 32). Its use in myasthenia gravis (MG), particularly in refractory disease, is expanding, with encouraging patient responses documented (33).

**Table 1:** Disease mechanistic basis and therapeutic strategies.

Autoimmune condition	Mechanism of autoimmunity	Major autoantibodies and targets	Non-B-cell therapies	B-cell-targeted therapies	References
Pemphigus vulgaris	Loss of cellular adhesion in epidermis of skin and mucosa leading to blistering	Anti-desmoglein 1/3	Corticosteroids, intravenous immunoglobulin, azathioprine, mycophenolate mofetil, cyclophosphamide, methotrexate, cyclosporine	Rituximab, ianalumab, DSG3-CAART cells, rilzabrutinib, tirabrutinib	(11–15)
Rheumatoid arthritis	Inflammation in joints caused by B and T cells and cytokine disruption (TNF-α, IL-6)	Anti-citrullinated protein antibodies, rheumatoid factor targeting Fc region of IgG	NSAIDs, corticosteroids, DMARDs, TNF inhibitors, JAK inhibitors	Rituximab, ofatumumab, belimumab, atacicept, tabalumab, ianalumab, telitacicept, rozibafusp alfa	(16, 17)
Sjögren’s syndrome	Abnormal accumulation of lymphocytes in exocrine glands; Hyperactivation of B cells and formation of ectopic germinal center	Anti-Ro/La targeting ribonucleoprotein complexes	Glucocorticoids, leflunomide, methotrexate, azathioprine, mycophenolate, or cyclophosphamide	Ianalumab, Belimumab, telitacicept, CAR-T cells, rituximab	(11, 18, 19)
Myasthenia gravis	Loss of neuromuscular transmission	Anti-AChR/MuSK targeting receptors at neuromuscular junction	Anticholinesterase inhibitors, corticosteroids, immunosuppressants, β-adrenergic agonists, azathioprine, tacrolimus, mycophenolate mofetil, methotrexate, cyclophosphamide, eculizumab, efgartigimod, intravenous immunoglobulin	Rituximab, inebilizumab, belimumab, telitacicept, CAR-T cells, MuSK-CAART cells	(11, 20, 21)
Systemic sclerosis	Vascular injury and overactivation of fibroblast causing fibrosis	Anti-topoisomerase I/centromere/RNA polymerase III	Mycophenolate mofetil, methotrexate, PDE5 inhibitors, tocilizumab, prostacyclin	Rituximab, inebilizumab, belimumab	(11, 22–24)

Therapies such as inebilizumab and obexelimab act against CD19, a surface marker present from early B-cell development through the plasmablast stage, thus targeting a broader segment of the B-cell lineage than CD20-directed mAb that targets mature B cells (34). In PV, inebilizumab has been associated with fewer blistering episodes and lower pathogenic autoantibody concentrations (35).

Bruton’s tyrosine kinase (BTK) inhibitors like fenebrutinib, rilzabrutinib, tirabrutinib, evobrutinib, and tolebrutinib disrupt intracellular signaling downstream of the B-cell receptor. By interrupting these pathways, they limit B-cell activation, clonal expansion, and antigen presentation without inducing full depletion (11, 36, 37). Initial clinical results point to reduced inflammatory activity and, in some cases, functional gains in RA, SSc, and pSS (36–39).

Furthermore, CAR T-cell therapy directed at CD19 is emerging as a potential option for severe autoimmune diseases unresponsive to conventional treatment (40). Pilot studies in systemic lupus erythematosus (SLE) and MG have reported rapid near-complete elimination of B cells, accompanied by long-lasting remission (41–43). Nonetheless, significant risks including cytokine release syndrome and neurotoxicity necessitate rigorous safety oversight (44).

Despite advancements in therapeutic strategies for autoimmunity, critical understanding of comparative B-cell therapeutics and their efficacy and durability across autoimmune conditions remains limited. Moreover, autoimmune conditions are mediated by different immune mechanisms and are not equally contributed by B cells, underscoring the need of disease-wise evaluation. Therefore, the objective of this review is to comprehensively assess efficacy and durability along with safety profiles of B-cell therapeutics such as B-cell depletion, B-cell signaling modulation, CAR-T cells, and combinatorial therapy across five different autoimmune conditions.

## Methods

This systematic review was conducted using PRISMA guidelines 2020 (45).

### Data sources and search strategy

For this review, a comprehensive literature search was performed using Embase, Web of Science, and PubMed databases for the articles published from 2020 to 2025 until July 2025. A filter including only randomized control trials, clinical trials, and studies was utilized during the process. The search strategy added in the **Supplementary Material** included the following listed MeSH terms and the keywords: B-cell-targeted therapies, B-cell therapies, anti cd20, anti cd19, RTX, ocrelizumab, ofatumumab, ublituximab, inebilizumab, B-cell trials, belimumab, and CART B cell (**Supplementary Table 1**). All these terms were used only after adding the autoimmune diseases under consideration such as B-cell-targeted therapies in PV, B-cell-targeted therapies in RA, and so on. The autoimmune diseases reviewed in this study are PV, RA, Sjögren’s syndrome (SS), SSc, and MG.

### Inclusion and exclusion criteria

The literature search was performed by two independent researchers to avoid bias and discrepancies while ensuring accuracy. We included only randomized controlled trials and clinical trials where the primary endpoint was assessing the effectiveness of B-cell therapy in humans suffering from the above-mentioned autoimmune disorders. We excluded non-human studies, animal studies, conference abstracts, case reports, observational studies, meta-analyses, and reviews.

### Data screening and inclusion

**Figure 2:**
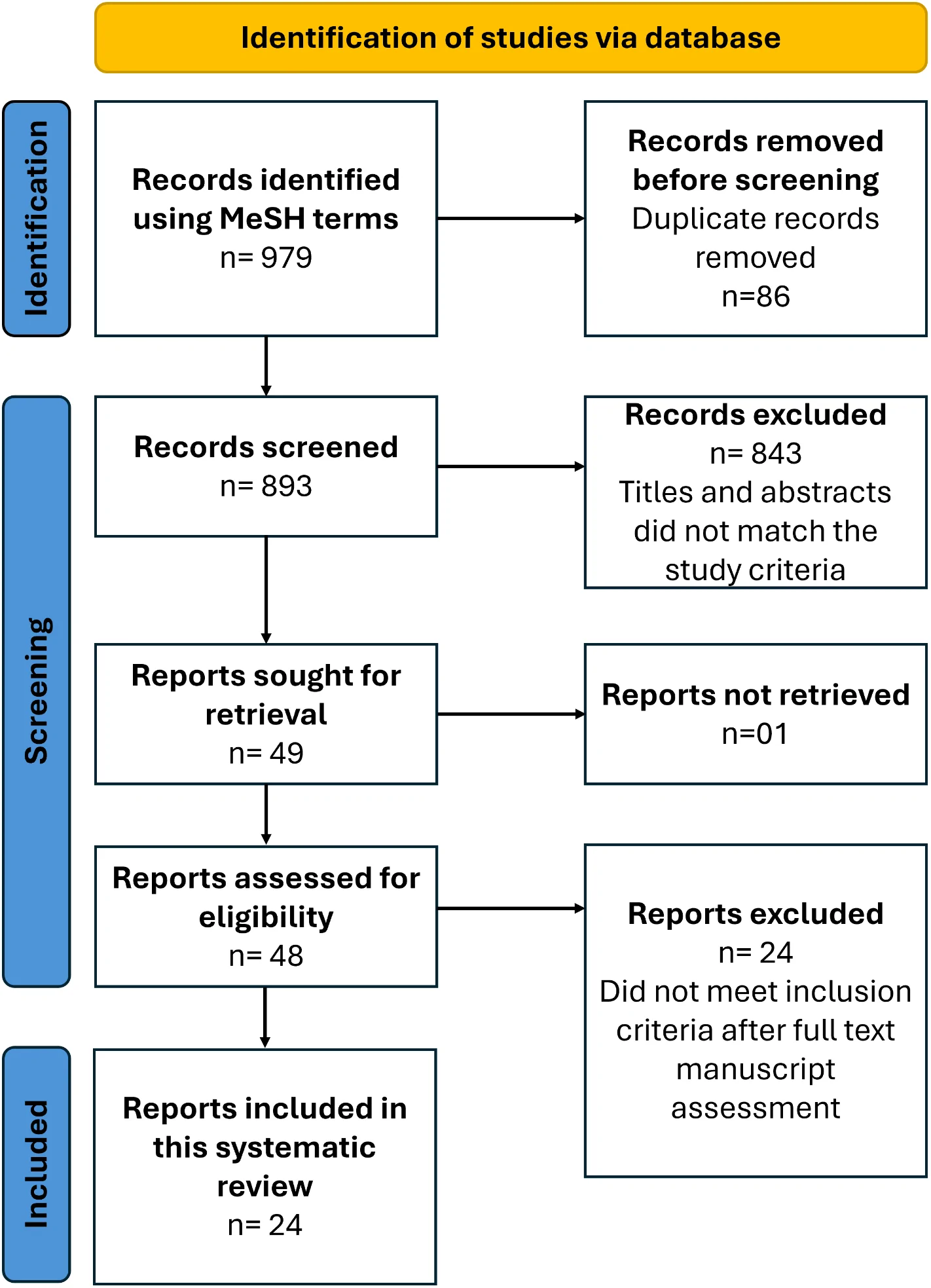
PRISMA flowchart mapping study selection process for systematic review.

Following the use of MeSH terms, a total of 979 studies were obtained, out of which 49 studies were initially found eligible for further screening. However, after employing the inclusion and exclusion criteria, 24 studies (5 PV, 13 RA, 1 SS, 2 SyS, and 3 MG) were found suitable to be included in this review (**Figure 2**). In our study, we included the baseline characteristics such as patient number, age, sex, study design, location, and prior therapies, and outcome data such as complete response, partial response, overall survival, primary endpoint, secondary endpoint, key result, and other complications.

### Risk of bias assessment

The quality of the included reports was assessed by two reviewers independently using the Cochrane Risk of Bias 2 (RoB2) tool and the ROBINS-I tool. The RoB2 tool assesses any bias across five domains: (D1) bias arising from the randomization process, (D2) bias due to deviations from intended interventions, (D3) bias due to missing outcome data, (D4) bias in measurement of the outcome, and (D5) bias in selection of the reported result, and then scored as low risk of bias, some concerns, or high risk of bias. An overall risk of bias further determines the quality of the study. ROBINS-I for non-randomized trials evaluate bias resulting from confounding factors, selection of study subjects, intervention, deviations from intended interventions, missing data, outcome measurement, and selection of reported results. We have harmonized and visually summarized the risk of bias assessment of both RoB2 and ROBINS-I using a unified traffic light plot for better comparison. Detailed information of RoB has been shared in the **Supplementary Material**.

## Results

This review evaluated 24 reports across five autoimmune conditions—PV, RA, MG, SSc, and SS.

### Risk of bias across studies

**Figure 3:**
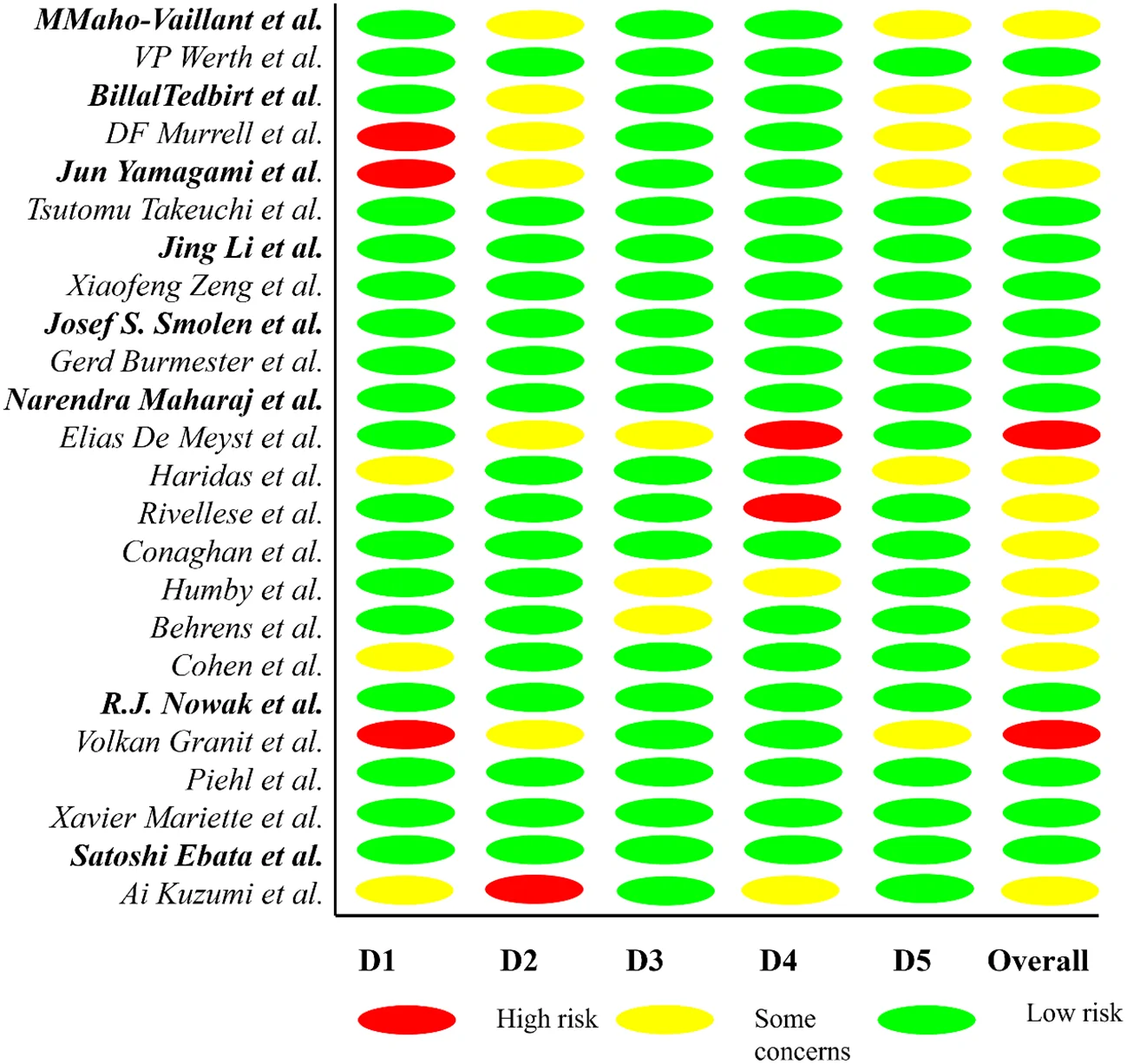
Risk of bias assessment. Traffic light plot presenting quality of reports included in the systematic review carried out using the Cochrane RoB2 tool and ROBINS-I. Red = high/serious risk, yellow = some concerns/moderate risk, and green = low risk. *n* = 24.

The risk of bias was evaluated using the Cochrane RoB 2 tool for randomized controlled trials and ROBINS-I for non-randomized single-arm trials. Out of 24 reports assessed, 11 were at low risk of bias, 11 studies had some concerns, and 2 studies were classified as high risk of bias (**Figure 3**). However, on individual domain assessment, D1, which is based on a randomization process that has inadequate information regarding randomization and concealment, demonstrated 18 studies with low risk of bias, 3 studies with some concern, and 3 non-randomized studies assessed by ROBINS-I with high risk due to absence of randomization and bias due to selection or confounding. D2 based on deviations demonstrated 5 studies with some concerns and 1 study with high risk due to the open-label study design and variable interventions while the remaining 18 studies were at low risk. D3 and D5 based on missing outcomes and selection of reported results showed few studies with some concerns while others with low risk. D4 based on outcome measures were also primarily low risk with two studies each of some concern and high risk. Overall RoB assessment suggests that most of the included reports were methodologically robust and with low risk or some concerns of bias.

### Pemphigus vulgaris

According to the inclusion and exclusion criteria for this review, five clinical studies examining B-cell-targeted treatments for PV were found suitable (46–50). This included two phase 2 single-arm studies and three phase 3 randomized controlled trials (**Table 2**). Studies were multicentric spanning Europe, USA, Japan, and Australia, and follow-up periods varied from 24 weeks to 7 years. Three phase 3 trials, a US-based randomized trial, and two RITUX3 trials directly compared RTX with common immunosuppressive treatments like mycophenolate mofetil (MMF) and prednisone, respectively (46–48). The remaining studies used open-label, uncontrolled designs to evaluate two novel BTK inhibitors—tirabrutinib and rilzabrutinib (49, 50).

**Table 2:** Characteristics of clinical trials evaluating B-cell-targeted therapy in autoimmune diseases.

Disease	Author (year)	Journal	Trial registration	Phase	Funding source	Study design	Intervention/comparator	Blinding	Follow-up duration	Location
Pemphigus vulgaris	Maho-Vaillant M et al. (2021) (46)	*J Invest Dermatol*	NCT00784589 (RITUX3)	Phase 3	Not reported	Randomized controlled trial (1:1)	Rituximab vs. prednisone	Open-label	36 months	Multicenter; France
	Werth VP et al. (2021) (47)	*N Engl J Med*	NCT02383589	Phase 3	Not reported	Randomized controlled trial (1:1)	Rituximab vs. mycophenolate mofetil	Double-blind, double-dummy	52 weeks	Multicenter; USA
	Tedbirt B et al. (2024) (48)	*JAMA Dermatol*	NCT00784589 (RITUX3)	Phase 3	French Society of Dermatology	Randomized controlled trial (1:1)	Rituximab vs. prednisone	Open-label	7 years	Multicenter; France
	Murrell DF et al. (2021) (49)	*Br J Dermatol*	NCT02704429	Phase 2	Principia Biopharma (Sanofi)	Single-arm (“BELIEVE” study)	Rilzabrutinib (single arm)	Not blinded	24 weeks	Multicenter; Australia, Croatia, France, Greece, Israel
	Yamagami J et al. (2021) (50)	*J Dermatol Sci*	NCT03762265	Phase 2	Ono Pharmaceutical (Japan)	Single-arm, uncontrolled	Tirabrutinib (single arm)	Not blinded	52 weeks	Multicenter; Japan
Rheumatoid arthritis	Takeuchi T et al. (2023) (51)	*Ann Rheum Dis*	NCT03605251	Phase 2	Taiho Pharmaceutical	Randomized, parallel-group	TAS5315 vs. placebo	Double-blind	12 and 36 weeks	Multicenter; Japan
	Li J et al. (2022) (52)	*Rheumatology (Oxford)*	NCT04192617	Phase 2	SinoMab BioScience	Randomized (1:1:1), multi-dose	SM03 (low/high dose) vs. placebo	Double-blind	24 weeks	Multicenter; China
	Zeng X et al. (2022) (53)	*Arthritis Res Ther*	NCT03522415	Phase 3	Shanghai Henlius Biotech	Randomized	HLX01 vs. placebo	Double-blind	48 weeks	Multicenter; China
	Smolen JS et al. (2020) (54)	*Rheumatology (Oxford)*	NCT01274182	Phase 2	Hexal AG; Sandoz	Randomized	Sandoz rituximab vs. reference RTX	Double-blind	24–52 weeks	Multicenter; Europe, USA, South America and Asia
	Burmester G et al. (2020) (55)	*Clin Pharmacol Drug Dev*	NCT02792699	Phase 3	Amgen Inc.	Randomized (1:1:1)	ABP 798 vs. RTX-US/EU	Double-blind	52 weeks	Multicenter; Europe and USA
	Maharaj N et al. (2024) (56)	*Arthritis Res Ther*	NCT0426877	Phase 3	Dr. Reddy’s Laboratories	Randomized	DRL_RI vs. RTX-US/EU	Double-blind	26 weeks	Multicenter; Europe and USA
	De Meyst E et al. (2024) (57)	*Trials*	NCT06003283	Not reported	Investigator-initiated	Parallel-group superiority RCT	Rituximab (dose-optimized, fixed-interval) vs. standard RTX	Open-label	104 weeks	Multicenter; Belgium
	Haridas et al. (2020) (58)	*BioDrugs*	NCT02296775	Phase 1/2	Dr. Reddy’s Laboratories Ltd.	Randomized, parallel-group	DRL RI (RTX biosimilar) vs. Rituxan (RTX‑US) vs. MabThera (RTX‑EU)	Double-blind	24 and 52 weeks	Multicenter; India and Ukraine
	Rivellese et al. (2023) (59)	*The Lancet Rheumatology*	2014-003529-16 (STRAP); 2017-004079-30 (STRAP-EU)	Phase 3	UK Medical Research Council and Versus Arthritis	Randomized (1:1:1), parallel-group	Rituximab vs. etanercept vs. tocilizumab	Open-label (blinded joint assessors and pathology review)	16 and 48 weeks	Multicenter; UK, Belgium, Italy, Portugal, and Spain
	Humby et al. (2021) (60)	*The Lancet*	ISRCTN97443826; EudraCT 2012-002535-28	Phase 4	UK-NIHR	Randomized controlled trial	Rituximab vs. tocilizumab	Open-label (blinded joint assessors)	48 weeks	Multicenter; UK, Belgium, Italy, Portugal and Spain
	Conaghan et al. (2023) (61)	*Lancet Rheumatology*	NCT02638948	Phase 2	Bristol Myers Squibb	Randomized, dose-ranging, placebo-controlled (1:1:1:1)	BMS-986142 100 mg vs. 200 mg vs. 350 mg vs. placebo	Double-blind	12 weeks treatment + 30-day follow-up	Multicenter; Europe, USA, South America, Asia, and Canada
	Behrens et al. (2021) (62)	*Rheumatology*	NCT01244958	Phase 3	Roche Pharma	Randomized, placebo-controlled trial	Rituximab + leflunomide vs. placebo + leflunomide	Double-blind	24 weeks	Multicenter; Germany
	Cohen et al. (2020) (38)	*Arthritis & Rheumatology*	NCT02833350	Phase 2	Genentech, Inc.	Randomized, placebo- and active-controlled trial	Fenebrutinib vs. placebo vs. adalimumab	Double-blind, double-dummy	12 weeks	Multicenter; Europe, Latin America and North America
Myasthenia gravis	Nowak RJ et al. (2025) (63)	*N Engl J Med*	NCT04524273	Phase 3	Amgen	Randomized, placebo-controlled	B-cell-targeted therapy vs. placebo	Double-blind	26 weeks	Multicenter; Japan and non-Japan
	Granit V et al. (2023) (64)	*Lancet Neurol*	NCT04146051	Phase 1b/2a	Cartesian Therapeutics; NINDS-NIH	Prospective, non-randomized	CAR-T cell therapy (single arm)	Open-label	9 months	Multicenter; USA
	Piehl et al. (2022) (65)	*JAMA Neurology*	NCT02950155	Phase 3	Swedish Medical Research Council	Randomized, placebo-controlled trial	Rituximab vs. placebo	Double-blind	48 weeks	Multicenter, Sweden
Sjögren’s syndrome	Mariette X et al. (2022) (66)	*JCI Insight*	NCT02631538	Phase 2	GSK	Randomized (1:2:2:2), multicenter	Placebo vs. belimumab + rituximab vs. belimumab vs. rituximab	Double-blind	52-week treatment + 16-week follow-up	Multicenter; Argentina, Canada, France, Germany, Italy, Netherlands, Norway, Spain, Sweden, UK
Systemic sclerosis	Ebata S et al. (2022) (67)	*Rheumatology*	NCT04274257	Phase 2	Not reported	Randomized, parallel-group, investigator-initiated	Rituximab vs. placebo	Double-blind	24 weeks	Multicenter; Japan
	Kuzumi A et al. (2023) (68)	*JAMA Dermatol*	NCT04274257	Phase 2/3	Not reported	Randomized double-blind open-label extension	Rituximab vs. placebo	Double-blind; open-label	24 weeks	Japan

Across studies, approximately 358 patients with age spanning from 18 to 80 years were assessed (**Table 3**). RTX reviewed in three studies consistently showed greater clinical efficacy when compared to other therapies (**Table 4**). As per the RITUX3 trial, 89% of the RTX-treated group demonstrated complete remission (CR) off therapy at 24 months compared to 34% in the prednisone group, with remission rates of 96% after 36 months (46). Additionally, the other phase 3 trial assessing RTX with MMF reported 40% of RTX-treated patients achieved remission compared to only 10% of the MMF group at 52 weeks, alongside reduction in steroid exposure (47). Treatment durability was also evaluated, evidenced by long-term follow-up data from the RITUX3 cohort that lasted up to 7 years (48). The 5-year disease-free survival (DFS) rates were 64.4% in RTX versus 23.3% in the prednisone group, and 7-year DFS rates were 62.2% in RTX versus 20.9% in the prednisone group. Across these RTX trials, significant reductions of anti-DSGs antibodies, DSG-specific B cells, T follicular helper cells, and IL-21 levels were also observed.

**Table 3:** Study population, eligibility criteria, and B-cell therapy specifications.

Disease	Author (year)	Inclusion criteria	Exclusion criteria	Total patients (per arm)	Age (years)	Sex	Severity	Prior therapies	B-cell therapy	Mechanism/Target	Dosage and schedule	Route
Pemphigus vulgaris	Maho-Vaillant M et al. (2021) (46)	18–80 years; new PV/PF; contraception	Pregnancy; HIV/HBV/HCV; Karnofsky <50%; cardiac disease; mAb allergy	90 (46 RTX/44 control)	Mean 53	Not reported	Moderate–severe	Not reported	Rituximab	Anti-CD20 B-cell depletion	1,000 mg d0 and d14; 500 mg at 12 and 18 months	IV
	Werth VP et al. (2021) (47)	18–75 years; moderate–severe PV; on steroids	Other blistering diseases; RTX ≤12 months	135 (67 RTX/68 MMF)	Median 48	66 F/59 M	Moderate–severe	Glucocorticoids	Rituximab	Anti-CD20	1,000 mg d1, d15, d168, d182	IV
	Tedbirt B et al. (2024) (48)	RITUX3 participants	As per RITUX3	90 (46/44)	~61	Not reported	Not reported	Not reported	Rituximab	Anti-CD20	As per RITUX3	IV
	Murrell DF et al. (2021) (49)	18–80 years; mild–severe PV; low-dose steroids	Malignancy; pregnancy; anti-CD20; infection	27	18–80	15 F/12 M	Moderate–severe	Low-dose steroids	Rilzabrutinib	BTK inhibitor	400 mg BID → 600 mg BID	Oral
	Yamagami J et al. (2021) (50)	≥20 years; refractory pemphigus; on steroids	Infection; immunosuppressants; BTKi	16	52.5 ± 8.8	8 F/8 M	Refractory	Steroids	Tirabrutinib	BTK inhibitor	80 mg once daily ×52 weeks	Oral
Rheumatoid arthritis	Takeuchi T et al. (2023) (51)	RA (ACR/EULAR); MTX-IR; ≥6 joints	Other DMARDs; TB; hepatitis; JAK/BTKi	91	20–64	65 F	Active RA	MTX	TAS5315	BTK inhibitor	2–4 mg once daily	Oral
	Li J et al. (2022) (52)	RA ≥12 months; MTX-IR; active disease	Other autoimmune disease; infection	156 (52/52/52)	18–75	Not reported	Active RA	MTX, NSAIDs, steroids	SM03	Anti-CD22 mAb	600 mg multidose	IV
	Zeng X et al. (2022) (53)	RA ≥6 months; MTX-IR	Other autoimmune disease; infection	275 (183/92)	Mean 49	233 F	Active RA	MTX	HLX01	RTX biosimilar (anti-CD20)	1,000 mg ×2	IV
	Smolen JS et al. (2020) (54)	RA; DMARD-IR	Infection; malignancy; hepatitis	290 (123/167)	Not reported	Not reported	Active RA	MTX, steroids	Sandoz RTX	RTX biosimilar (anti-CD20)	1,000 mg ×2, 2 weeks apart	IV
	Burmester G et al. (2020) (55)	RA; MTX-IR; active disease	Prior RTX; Felty syndrome	311 (104/104/103)	Mean 55.9	~90 F/group	Active RA	MTX	ABP-798	RTX biosimilar (anti-CD20)	2 cycles of 1,000 mg ×2	IP
	Maharaj N et al. (2024) (56)	Prior RTX responders; MTX stable	Other biologics; JAK inhibitors	140 (70/70)	Mean 59.8	115 F/25 M	Active RA	MTX, steroids	DRL-RI	RTX biosimilar (anti-CD20)	1,000 mg d1 and d15	IV
	De Meyst E et al. (2024) (57)	RA; prior RTX response	Non-RTX DMARDs	134	Not reported	Not reported	Active RA	csDMARDs	Rituximab	Anti-CD20	1,000 mg every 24 weeks	IV
	Haridas et al. (2020) (58)	18–65 years, RA, MTX-IR, biologic‑naïve	Systemic RA, HIV, confounding diseases	276 (91/92/93)	Mean 44	~88% F	Moderate–severe active RA	MTX, stable steroids	DRL-RI	RTX biosimilar (anti-CD20)	1,000 mg d1 and d15	IV
	Rivellese et al. (2023) (59)	≥18 years, RA (ACR/EULAR); MTX or DMARD-IR	Steroid injection, uncontrolled disease	226 (79/73/72)	Mean ~52	75% F	Moderate–severe active RA	csDMARDs	Rituximab	Anti‑CD20	1,000 mg weeks 0 and 3	IV
	Humby et al. (2021) (60)	≥18 years, RA by ACR/EULAR, anti‑TNF- IR	Other infection or comorbidity, biopsy unsuitable	164 (83/81)	Mean 57	~70% F	Active RA	csDMARDs + ≥1 TNFi	Rituximab	Anti-CD20	1,000 mg ×2, 2 weeks apart	IV
	Conaghan et al. (2023) (61)	≥18 years, RA (ACR/EULAR); MTX or TNF-IR	Juvenile RA, Felty syndrome, infection	248 (75/73/73/26)	Mean 56.7	214/247 (87%) female	Moderate-to-severe active RA	MTX	BMS-986142	BTK inhibitor	100 mg, 200 mg, or 350 mg OD	Oral
	Behrens et al. (2021) (62)	18–75 years, RA >3.2, leflunomide (LEF) IR	DMARDs, biologics other than TNFi, infections	140 (93/47)	Mean 57	Not reported	Moderate–severe	Prior csDMARDs, ≤2 TNFi	Rituximab	Anti‑CD20	1,000 mg d1 and d15 + LEF	IV
	Cohen et al. (2020) (38)	18–75 years, RA, ACR/EULAR, MTX‑IR (cohort1) or TNF‑IR (cohort2)	Other autoimmune disease, prior BTK/JAK/B-cell therapy	578 (480/98)	Mean 55	~80% female	Moderate–severe	MTX‑IR or TNF‑IR	Fenebrutinib	BTK inhibitor	50 mg QD/150 mg QD/200 mg BID	Oral
Myasthenia gravis	Nowak RJ et al. (2025) (63)	AChR/MuSK+; MGFA II–IV; MG-ADL ≥6	Recent cyclosporine/tacrolimus/methotrexate; high-dose steroids	238 (119/119)	47.5 ± 15.3	144F	MGFA II–IV	Steroids, AZA, MMF	Inebilizumab	Anti-CD19	Every 6 months	IV
	Granit V et al. (2023) (64)	MGFA II–IV; antibody+; MG-ADL ≥6	Uncontrolled disease; recent IVIG/PLEX	14	52	10 F/4 M	MGFA II–IV	Steroids, AZA, MMF	Descartes-08	Anti-BCMA CAR-T	Escalating IV infusions	IV
	Piehl et al. (2022) (65)	≥18 years, MG, QMG ≥6, MGFA II–IV	Thymoma, thymectomy, prior immunosuppressants	47 (25/22)	Mean 62	Not reported	MGFA II–IV	Steroids, immunosuppressants	Rituximab	Anti‑CD20	Single 500 mg	IV
Sjögren’s syndrome	Mariette X et al. (2022) (66)	AECG criteria; anti-SSA/SSB+; ESSDAI ≥5	Secondary SS; malignancy; infection; prior B-cell depletion	86 (4 arms) 60 completed till week 68	45–55	80 F/6 M	Systemic pSS	Steroids, immunomodulators	Belimumab ± RTX	Anti-BLyS ± anti-CD20	Protocol-defined	IV
Systemic sclerosis	Ebata S et al. (2022) (67)	SSc (2016); pred ≤10 mg/day	Pulmonary HTN; malignancy; infection	56	48 (12–24, 40–78)	49 F/7 M	mRSS ≥2	Low-dose steroids	Rituximab	Anti-CD20	375 mg/m^2^ weekly ×4	IV
	Kuzumi A et al. (2023) (68)	SSc (ACR/EULAR); mRSS ≥10	Pulmonary HTN; cyclophosphamide	56 DB; 46 OLE	Median 48	27 F/2 M	Moderate–severe	Supportive	Rituximab	Anti-CD20	375 mg/m^2^ weekly ×4 q6mo	IV

PV, pemphigus vulgaris; PF, pemphigus foliaceus; HIV, human immunodeficiency virus; HBV, hepatitis B virus; HCV, hepatitis C virus; MAB, monoclonal antibody; IV, intravenous; BTK, Bruton’s tyrosine kinase; DMARDs, disease-modifying anti-rheumatic drugs; TB, tuberculosis; JAK, Janus kinase; ACR, American College of Rheumatology; EULAR, European Alliance of Associations for Rheumatology; MTX, methotrexate; RA, rheumatoid arthritis; NSAID, non-steroidal anti-inflammatory drug; AZA, azathioprine; MMF, mycophenolate mofetil; MUSK, muscle-specific kinase; MGFA, Myasthenia Gravis Foundation of America; MG-ADL, Myasthenia Gravis–Activities of Daily Living; BCMA, B-cell maturation antigen; CAR-T, chimeric antigen receptor T cell; IVIG, intravenous immunoglobulin; PLEX, plasma exchange; AECG, American–European Consensus Group; SSA/B, Sjögren’s syndrome–related antigen A/B; ESSDAI, European League Against Rheumatism Sjögren’s Syndrome Disease Activity Index; SS, Sjögren’s syndrome; HTN, hypertension; SSC, systemic sclerosis; MRSS, Modified Rodnan Skin Score.

**Table 4:** Clinical, laboratory, and safety outcomes of B-cell-targeted therapies.

Disease	Author (year)	Primary endpoint	Secondary endpoints	Efficacy outcomes	Laboratory outcomes	Safety outcomes
Pemphigus vulgaris	Maho-Vaillant M et al. (2021) (46)	Complete remission off therapy at month 24	CR on minimal therapy; time to CR; relapse; cumulative steroid dose; QoL	CR off therapy: 89% RTX vs. 34% control at 24 months; 96% RTX at 36 months; marked steroid reduction	↓ Anti-DSG1/3 IgG, disappearance of DSG-specific B cells, ↓ TFH cells, ↓ IL-21	No unexpected adverse events
	Werth VP et al. (2021) (47)	Complete remission at week 52	Steroid dose; disease flares; DLQI	Sustained remission: 40% RTX vs. 10% MMF; DLQI −8.87 vs. −6; lower steroid exposure	Greater ↓ anti-DSG3 in RTX arm; transient T-cell reduction	AEs: 85% RTX vs. 88% MMF; SAEs: 22% vs. 9%; 1 death (MMF)
	Tedbirt B et al. (2024) (48)	5- and 7-year disease-free survival (DFS)	Overall relapse rate; SAEs	5-year DFS: 64.4% RTX vs. 23.3% prednisone; relapse 42.2% vs. 83.7%; 7-year DFS: 62.2% vs. 20.9%, first-line RTX superior	Anti-DSG1/3 negativity maintained in CR; antibody thresholds predicted relapse	Fewer SAEs with RTX; 1 death per arm
	Murrell DF et al. (2021) (49)	Disease control at 4 weeks with low/no steroids	CR, PDAI, relapse, QoL, PK/PD	CDA by week 4: 52%; CR 22% by week 24; rapid PDAI improvement; steroid sparing	↓ Anti-DSG3 IgG; BTK occupancy in PBMCs	TEAEs 74%; no major safety signals
	Yamagami J et al. (2021) (50)	CR off therapy at month 24	Remission/CR over time; PDAI; antibodies; steroid dose	Week 52: CR 50%, remission 64%; PDAI and steroid dose markedly reduced	↓ Anti-DSG1/3; ↓ CD19+ B cells; IgM ↓ 26%; IgG stable	AEs 87.5%; SAEs 18.8%; discontinuation 12.5%
Rheumatoid arthritis	Takeuchi T et al. (2023) (51)	ACR20 at week 12	ACR50/70; DAS28; HAQ-DI; biomarkers	ACR20: 78.9% TAS5315 vs. 60% placebo; ACR50: 33.3% TAS5315 vs. 13.3% placebo; ACR70: 7% TAS5315 vs. 0% placebo; improved SDAI/CDAI, DAS28, HAQ-DI	↓ IgG, IgM, RF, ACPA greater vs. placebo	AEs 41%–45%; 1 drug-related SAE; no dose dependency
	Li J et al. (2022) (52)	ACR20 at week 24	ACR50/70; DAS28; HAQ-DI	ACR20: 65.3% high-dose, 56.9% low-dose vs. 34% placebo; DAS28 and HAQ-DI significantly improved	↓ CD19+ B cells (dose-dependent)	AEs 40.6%; SAEs rare; no deaths
	Zeng X et al. (2022) (53)	ACR20 at week 24	ACR50/70; DAS28; HAQ-DI; PROs	ACR20: 60.3% HLX01 vs. 37.1% placebo; higher remission and LDA rates	Greater ↓ CRP and ESR; low ADA incidence	AEs ~83%; SAEs ~7%; no deaths
	Smolen JS et al. (2020) (54)	PK equivalence (AUCinf) at week 24	DAS28; ACR responses; B-cell depletion	Similar DAS28 (~3.3) and ACR20/50/70 up to week 52; sustained disease control	Comparable B-cell depletion; similar ADA rates	Mild/moderate AEs; low SAE incidence
	Burmester G et al. (2020) (55)	PK equivalence (AUCinf, Cmax)	DAS28; ACR; CD19 depletion	Equivalent PK/PD and disease control across biosimilars	Rapid and similar CD19 depletion; comparable immunogenicity	≥Grade-3 AEs 3%–6%; few discontinuations
	Maharaj N et al. (2024) (56)	ADA incidence	ADA titers; neutralizing antibodies; PK	Comparable immunogenicity and drug levels between biosimilar and RTX	Low ADA/NAb incidence; no PK impact	TEAEs ~35%; mild infusion reactions only
	De Meyst E et al. (2024) (57)	RAID score AUC over 104 weeks	DAS28; SDAI; steroid dose; tapering success	Maintained disease control; successful RTX tapering in subset	CD19 depletion; monitoring of Ig levels	Long-term safety; serious infections monitored
	Haridas et al. (2020) (58)	PK similarity (AUC 0-14days, first infusion and AUCinf, entire course)	Cmax; DAS28‑CRP; ACR20/50/70;PK/PD, HAQ‑DI; B-cell depletion/recovery	PK endpoints within 80%–125% equivalence; comparable efficacy across arms	Peripheral B cell ↓; ADA ↓	Comparable AE rates across arms
	Rivellese et al. (2023) (59)	ACR20 at week 16	ACR50/70; DAS28 remission; CDAI	No difference: 60% ETN/TCZ vs. 59% RTX in B-cell-poor; structural progression higher in B-cell-rich RTX group	Synovial biopsy: B-cell-poor vs. -rich signatures; RNAseq pathotypes	SAEs: 6% RTX, 6% ETN, 4% TCZ; no major differences
	Humby et al. (2021) (60)	CDAI50% at week 16	CDAI‑MTR; DAS28; patient-reported outcomes	Histology: no difference; RNAseq: TCZ superior (63% vs. 36% CDAI50%)	RNAseq B-cell signature stratification predictive	AEs: 70% RTX vs. 80% TCZ; SAEs: 7% vs. 10%; no significant difference
	Conaghan et al. (2023) (61)	ACR20/70 at week 12	ACR50, DAS28-CRP/ESR, CDAI, SDAI	Co-primary endpoints were not met. ACR20/70 responses not significant	↓ CXCL13, ↓ plasma-cell genes, ↓ IgA, IgG and IgM	100–200 mg well tolerated; AEs comparable
	Behrens et al. (2021) (62)	ACR50 at week 24	ACR20/70, DAS28, CDAI, HAQ‑DI, QoL	ACR50: 27% RTX+LEF vs. 15% Placebo +LEF; ACR20 significant at weeks 12–24; DAS28 remission 28% vs. 6%	↓ CD19+/CD20+ B cells	AE rates similar (71% vs. 70%); SAEs higher with RTX+LEF (20% vs. 2%)
	Cohen et al. (2020) (38)	ACR50 at week 12	ACR20/70, DAS28, HAQ‑DI, CRP, ESR	ACR50: 35% Fenebrutinib 200 mg vs. 15% placebo; comparable to ADA (36%)	↓ RF autoantibody, ↓ CCL4, ↓ TL1A, ↓ IL‑6	Common AEs
Myasthenia gravis	Nowak RJ et al. (2025) (63)	Change in MG-ADL at week 26	QMG; responder rate; steroid tapering	MG-ADL −4.2 vs. −2.2 placebo (*p* < 0.001); greater reduction in QMG score; sustained benefit	Rapid and sustained B-cell depletion	AEs 80.7% vs. 73.1%; SAEs fewer with inebilizumab
	Granit V et al. (2023) (64)	Safety and tolerability	MG-ADL; QMG; MGC; QoL	MG-ADL (−6), OMG (−7) improvements; reduced IVIG requirement	↓ Anti-AChR; ↓ BAFF/APRIL; CAR-T expansion	No DLTs; mainly mild AEs
	Piehl et al. (2022) (65)	Minimal disease manifestations at week 16	QMG; MG‑ADL; MG‑QoL; rescue therapy use	71% RTX vs. 29% placebo; fewer rescue therapies (4% vs. 36%)	No significant change in AChR titers; trend ↓ with RTX	AE rates higher RTX (81 vs. 44 events); SAEs: 6 vs. 4; one fatal cardiac event in RTX arm
Sjögren’s syndrome	Mariette X et al. (2022) (66)	Safety through week 68	ESSDAI; ClinESSDAI; salivary flow; ESSPRI	Greater ESSDAI reduction with belimumab + RTX; improved salivary flow	Near-complete B-cell depletion; ↓ CXCL13; delayed repopulation	AE rates similar; SAEs higher in active arms; 1 unrelated death
Systemic sclerosis	Ebata S et al. (2022) (67)	Change in mRSS at 24 weeks	Subgroup analyses (CD19, SP-D)	Greatest benefit in high-CD19 and high-mRSS patients	Baseline CD19 and mRSS predicted response	Well tolerated; no unexpected signals
	Kuzumi A et al. (2023) (68)	Change in mRSS at 24 weeks	FVC%; biomarkers; responder analysis	Significant skin improvement; gradual FVC% gain	Rapid CD19/CD20 depletion; ↓ KL-6; ↓ IgA/IgG correlated with response	AEs 93% (mostly mild); 1 unrelated SAE

ACPA, anti–citrullinated protein antibody; ADA, anti-drug antibody; AE, adverse event; APRIL, A proliferation-inducing ligand; AUCINF, area under the concentration–time curve to infinity; BAFF, B-cell activating factor (B lymphocyte stimulator; BLyS); CDAI, Clinical Disease Activity Index; CLINESSDAI, Clinical European League Against Rheumatism Sjögren’s Syndrome Disease Activity Index; CR, complete remission; CRP, C-reactive protein; DAS, Disease Activity Score; DFS, disease-free survival; DLQI, Dermatology Life Quality Index; DLTS, dose-limiting toxicities; DSG, desmoglein; FVC, forced vital capacity; HAQ-DI, Health Assessment Questionnaire–Disability Index; IR, infusion reaction; LDA, low disease activity; MG, myasthenia gravis; NAB, neutralizing antibody; NR, not reported; PDAI, Pemphigus Disease Area Index; PK/PD, pharmacokinetics/pharmacodynamics; PROS, patient-reported outcomes; QMG, Quantitative Myasthenia Gravis Score; QOL, quality of life; RAID, rheumatoid arthritis impact of disease; RF, rheumatoid factor; SAEs, serious adverse events; SDAI, Simplified Disease Activity Index; SP-D, surfactant protein D; TEAE, treatment-emergent adverse event; TFH, T follicular helper cell.

↓, decreased/ downregulated.

Unlike anti-CD20 RTX used in other trials for B-cell depletion, subsequent studies used an alternative approach to suppress pathogenic B-cell signaling via BTK inhibitors. Rilzabrutinib achieved CR in 22% cases at week 24 with disease activity control in 52% by week 4, along with steroid-sparing effects (49). Tirabrutinib, on the other hand, resulted in CR in 50% cases with overall remission in 64% cases by week 52 (50). Like RTX, BTK inhibition demonstrated reduction in anti-DSG antibodies and CD19 B cells, along with selective reduction in IgM levels.

The safety profile of these interventions across trials suggested that RTX has fewer serious adverse events (SAEs) in the long run and is generally well tolerated. Furthermore, SAEs also occurred rarely for BTK inhibitors; however, several mild to moderate adverse effects were seen, without major safety concerns.

### Rheumatoid arthritis

There were 13 studies on B-cell-targeted therapies in RA that included six phase 2 trials, five phase 3 trials, one phase 4 trial, and a 104-week-long investigator-initiated trial (38, 51–62). These trials were randomized and were mostly double-blind except for three that were open-label. All the studies were performed across multiple centers either in a single country or globally spanning Asia, Europe, South America, UK, Canada, and the USA (**Table 2**). Interventions targeted by the trials include BTK inhibition, anti-CD22, RTX, and its biosimilars, assessing over 3,000 patients across trials. The patients were mainly middle-aged with age ranging from 18 to 80 years, mostly women, having active RA despite methotrexate, leflunomide, or previous disease-modifying anti-rheumatic drug (DMARD) therapies (**Table 3**).

In placebo-controlled trials, BTK inhibition and anti-CD22 therapy had much greater improvement in clinical disease activity and ACR20 response rates, 78.9% vs. 60% by BTK inhibition at week 12 and 65.3% vs. 34% at week 24 by anti-CD22 therapy accompanied by improvement in DAS28 and HAQ-DI scores (**Table 4**) (51, 52). HLX01 biosimilar also showed robust activity with ACR20 response rate 60.3% vs. 37.1% in placebo and greater remission (53). However, other biosimilar equivalence trials continued to show similar pharmacokinetic properties and clinical efficacy, and the same response duration in RTX biosimilars and the reference product (54–56, 58). Prolonged post follow-up might aid in long-term disease management and indicated that RTX tapering in the dose could be a possibility in certain patients without losing clinical efficacy (57). RTX and leflunomide dual therapy demonstrated greater improvement in disease activity as well as ACR50 responses (62). Furthermore, 200 mg of fenebrutinib displayed comparable ACR50 response to adalimumab (35%) along with improvement in DAS28, CDAI, and SDAI scores (69). Biopsy-guided studies by Humby et al. and Rivellese et al. highlighted that therapeutic responses varied depending on the synovial B-cell profiling. B-cell-rich diseases showed greater improvement on treatment with RTX, whereas B-cell-poor diseases displayed benefits on treatment with tocilizumab. Additionally, RNA-sequencing-based profiling of B-cell signatures was more predictive of therapeutic outcomes when compared to histology alone (59, 60).

Laboratory results showed that there was rapid and persistent B-cell depletion (CD19-positive), decreased rheumatoid factor and anti-citrullinated protein antibodies, and elevated inflammatory markers (CRP and ESR). Based on biosimilar studies, similar immunogenicity and pharmacodynamic characteristics were evident with reference RTX. In all the RA trials, the therapeutic interventions were well tolerated with the majority of adverse events being mild to moderate and SAEs being rare.

### Myasthenia gravis

Three trials explored B-cell-targeted treatment in generalized MG (63–65). These trials included two phase 3 randomized trials, one compared the anti-CD19 antibody inebilizumab and another compared RTX to placebo, and a phase 1b/2a open-label phase analysis, which compared BCMA-directed CAR-T cell therapy (Descartes-08) (**Table 2**). These were multicenter studies that included patients with antibody-positive disease of MGFA with a II–IV level and severe functional impairment (**Table 3**). Together, these trials targeted both traditional antibody-mediated B-cell depletion by inebilizumab and novel next-generation therapy, i.e., CAR-T cell therapy for depletion of long-lived plasma cells. The inebilizumab trial recruited 238 patients, the RTX trial enrolled 47 patients, and the CART trial, being small and early phase, included only 14 patients.

Inebilizumab showed a much higher effect on improving MG-ADL scores than placebo (−4.2 vs. −2.2) in the phase 3 trial and had benefits on quantitative scores of MG and reduced steroid requirements (**Table 4**). Furthermore, pronounced and persistent reduction of peripheral B cells was observed (63). In the same way, RTX improved quantitative scores and demonstrated increased efficacy of 71% compared to 29% of placebo (65). In the CAR-T trial, early indicators of clinical efficacy, such as functional outcome improvements in numeric scores of MG-ADL and QMG and decreased intravenous immunoglobulin dependency, were reported (64). Additionally, CAR-T expansion, anti-acetylcholine receptor antibodies reduction, and BAFF/APRIL reduction were observed post therapy, supporting the sustained depletion of B cells. Overall, adverse events were mainly mild and there were no dose-limiting toxicity in the CAR-T trial and the inebilizumab had a similar safety profile to the placebo.

### Sjögren’s syndrome

A single randomized multicenter phase 2 trial compared B-cell targeting with belimumab, RTX, or a combination of both belimumab and RTX to placebo in pSS (66). A total of 86 patients were assessed, with 60 patients completing 16 weeks of follow-up post 52 weeks of treatment. The mean age range was 45 to 55 years with predominantly female patients (**Table 3**). The patients were systemically diseased and had been previously exposed to immunomodulatory therapy. The clinical efficacy across groups through week 68 demonstrated that the interaction of belimumab and RTX generated the largest decrease in the systemic disease activity scores (ESSDAI) and salivary gland functioning in comparison to monotherapy and placebo (**Table 4**). Furthermore, this study revealed an almost total loss of B cells, a delay in repopulation, and lower CXCL13 and other B-cell activation biomarkers after combination therapy. The adverse event rates were similar when compared across the groups, but SAEs were higher in active treatment arms specifically in the combination group, and no regular new safety issues were detected.

### Systemic sclerosis

Two randomized trials were found suitable and were included in this review (67, 68). They included a double-blind placebo-controlled phase 2 trial and a double-blind, open-ended phase 2/3 study, both assessing RTX in SSc. Both trials were conducted in Japan (**Table 2**). The median age was 48 years with primarily female patients. Both trials had a follow-up duration of 24 weeks (**Table 3**).

The skin involvement was moderate to severe, and there were limited treatment options in patients. Both trials reported significant reduction in modified Rodnan Skin Scores (mRSS) after 24 weeks in the RTX group when compared to placebo. Ebata et al. showed that patients who had higher initial counts of CD19-positive B cells, as well as more severe skin disease, had the highest clinical benefit and improvement in thickness of the skin (67). Kuzumi et al. reported enhancement in parameters of pulmonary functioning over time (**Table 3**) (68).

Additionally, the rate of depletion of CD19/CD20-positive B cells was rapid, and the changes in immunoglobulins and clinical response correlated. Depleting KL-6 levels in serum with decreased IgA and IgG were also associated with improved skin scores. RTX was usually well tolerated, and most of the adverse events were mild and no safety signals were unexpectedly observed. However, Kuzumi et al. reported at least one adverse event happened in 93% of patients, which was generally mild in nature (**Table 4**).

### Ongoing clinical trials in pemphigus vulgaris, rheumatoid arthritis, myasthenia gravis, systemic sclerosis, and Sjögren’s syndrome

The current scenario of the ongoing clinical trials highlights a clear transition towards the modern next-generation engineered strategies from the conventional B-cell therapies. A search through the ClinicalTrial database suggests a total of 72 unique ongoing trials in these five autoimmune conditions (**Table 5**) (70). SSc has the greatest number of ongoing trials (*n* = 19), followed by MG (*n* = 18), SS (*n* = 6), and PV (*n* = 3). RA has no disease-specific B-cell ongoing trial; however, many multi-disease trials have included it. There are 26 ongoing trials in multi-autoimmune diseases that included study participants from more than one autoimmune condition, reflecting an approach to utilize immunotherapy to diseases sharing common pathologies. Overall, CAR-based strategies including CD19 or dual CD19-BCMA dominated the current clinical landscape. Other strategies included CD20 CART or CAR NK and BAFF/APRIL-directed B-cell therapies. Most trials are in phase 1 or phase 1/2, suggesting early stages of the next-generation clinical development.

**Table 5:** Ongoing B-cell-directed therapy trials in autoimmune diseases.

Disease	NCT ID	Investigational therapy	Target/Mechanism	Phase	Estimated completion
Rheumatoid arthritis	*No specific ongoing trial; only included in multi-disease trials*				
Pemphigus vulgaris	NCT04422912	DSG3-CAART/CABA-201 (resecabtagene autoleucel)	CAAR-T cells targeting DSG3 autoreactive B cells/CD19 CAR-T	1/2	2029
	NCT07681388	CAR-T	CD19 CAR-T	NA	2028
	NCT06663943	CM313	CD38	1/2	2026
Myasthenia gravis	NCT06371040	CD19/BCMA-targeted CAR-T therapy	CD19/BCMA CAR-T	1	2026
	NCT07243366	Allogeneic CD19 CAR-NK cells	CD19 CAR-NK	1	2027
	NCT06933563	UCAR-T	CD19/BCMA CAR-T	1	2027
	NCT06193889	KYV-101	CD19 CAR-T	2	2028
	NCT06359041	CABA-201	CD19 CAR-T	1/2	2029
	NCT06220201	CC-97540 (BMS-986353)	CD19 NEX-T CAR-T	1	2027
	NCT07304154	KITE-363	Dual-target CD19/CD20 CAR-T	1	2029
	NCT07596901	Aritinercept	Dual BAFF/APRIL	1/2	2029
	NCT04146051	Descartes-08 CAR-T	BCMA	2	2026
	NCT06939166	UCAR-T	CD19/BCMA CAR-T	1	2027
	NCT06688435	SYS6020 CAR-T	BCMA	1	2033
	NCT07337785	RD06-05 Universal CAR-T	CD19/BCMA CAR-T	1	2028
	NCT05868837	Rituximab	CD20	3	2025
	NCT06342544	Rituximab	CD20	3	2028
	NCT07071246	Rituximab	CD20	NA	2027
	NCT07556120	HN2301 *in vivo* CAR-T	CAR-T	1	2027
	NCT07058298	GC012F CAR-T	CD19/BCMA	1	2027
	NCT06626919	Anitocabtagene autoleucel (anito-cel; CART-ddBCMA)	BCMA	1	2028
Sjögren’s syndrome	NCT06056921	CD19 CAR-T cells	CD19	1	2026
	NCT06420154	CD19 CAR-T cells	CD19	1	2027
	NCT07041099	CLN-978 CD19-directed T-cell engager	CD19/CD3	1	2029
	NCT07371468	GSK5926371	Investigational B-cell-targeted therapy	1	2028
	NCT05349214	Ianalumab (VAY736)	BAFF-R (CD268)	3	2027
	NCT07621809	Ianalumab (VAY736)	BAFF-R (CD268)	3	2033
Systemic sclerosis	NCT06655896	Rapcabtagene autoleucel versus rituximab	CD19 CAR-T	2	2032
	NCT03844061	Belimumab + rituximab	BAFF + CD20	2	2026
	NCT07335562	Zolacabtagene autoleucel (BMS-986353, Zola-cel)	CD19 CAR-T	3	2030
	NCT06549231	Rituximab + mycophenolate mofetil (MMF)	CD20	3	2029
	NCT07305116	Universal allogeneic UCAR T-cell therapy	CD19/BCMA CAR-T	1	2029
	NCT06255028	CNTY-101 (allogeneic iPSC-derived CAR-NK cell therapy)	CD19	1	2028
	NCT06308978	FT819 (iPSC-derived allogeneic CAR-T cell therapy)	CD19	1	2042
	NCT07339540	V001-BCMA (*in vivo* LV gene therapy)	BCMA	1	2028
	NCT06947460	CD19/BCMA CAR-T cell therapy	CD19/BCMA CAR-T	1	2026
	NCT07315087	QT-019C	CD19/BCMA CAR-T	1	2029
	NCT06941129	UCAR-T	CD19/BCMA CAR-T	1	2028
	NCT07490275	CAR-γδT cell therapy	CD19/BCMA CAR-T	1	2028
	NCT07586267	QT-219CX (CD19/BCMA UCAR-T)	CD19/BCMA CAR-T	1	2030
	NCT07674147	RD06-05	CD19/BCMA CAR-T	1	2029
	NCT07507201	QT-219C	CD19/BCMA CAR-T	1	2029
	NCT07155369	UCAR-T cells	CD19/BCMA CAR-T	NA	2028
	NCT06375005	Telitacicept	Dual BAFF/APRIL	2	2027
	NCT06843239	Tibulizumab (ZB-106)	Dual BAFF/IL-17A	2	2028
	NCT06801119	HN2301	CAR-T	1	2028
Trials occurring in multiple autoimmune diseases					
RA, PV	NCT06581562	AB-101 + rituximab	NK-cell + CD20	I	2028
RA, SyS	NCT07558850	Anti-CD19/BCMA universal CAR-T (UCAR-T)	CD19/BCMA CAR-T	NA	2029
	NCT07295847	AZD0120 (GC012F)	CD19/BCMA CAR-T	1	2028
RA, SyS, SS	NCT07121777	LCAR1901	CD19/BCMA CAR	NA	2029
	NCT07246096	KN3601	CD19/BCMA CAR-T	1	2028
	NCT06503224	SCAR02	CD19/BCMA CAR-T	NA	2028
	NCT07052565	ECAR01	CD19/BCMA CAR-T	NA	2028
	NCT06428188	Sequential CAR-T	CD19/BCMA CAR-T	1/2	2026
	NCT06991114	Allogeneic NK + rituximab	NK/CD20	2	2029
RA, MG, SyS	NCT07085676	HBI0101 CAR-T	BCMA CAR-T	1	2030
RA, SyS, SS, MG	NCT06548607	CD19 or CD19/BCMA CAR-T therapy (BHCT-RD06)	CD19 or CD19/BCMA	1	2027
	NCT07361094	Autologous CD19/BCMA dual-target CAR-T	CD19/BCMA CAR-T	1	2028
MG, SyS	NCT07676266	C-CAR168	CD20/BCMA CAR-T	1	2028
	NCT06249438	C-CAR168	CD20/BCMA CAR-T	1	2040
	NCT06775912	RD06-05	CD19/BCMA CAR-T	1	2027
SyS, SS	NCT07212322	ET-902-AID01 universal CAR-T	CD19	NA	2027
	NCT07301164	BCT301 CAR-iT	CD19	1	2028
	NCT07413341	TI-0032-III	CD19	1	2027
	NCT07184450	BCMA/CD70-targeted CAR-T therapy	BCMA and CD70	1	2028
	NCT06947473	Umbilical cord blood CD19-BCMA CAR-T cell therapy	CD19/BCMA CAR-T	1/2	2027
	NCT06350110	BH002 CAR-T	CD19/BCMA CAR-T	1/2	2025
	NCT06821659	UWD-CD19 universal CAR-T	CD19	1/2	2028
	NCT06828042	QH103 universal CAR-γδT cells	CD19	1/2	2027
	NCT06794008	BCMA/CD19 CAR-T	CD19/BCMA CAR-T	2	2027
	NCT07596680	RD06-05	CD19/BCMA CAR-T	1	2029
MG, SyS, SS	NCT06978738	UCAR-T	CD19/BCMA CAR-T	1	2028

## Discussion

This systematic review is based on assessing and synthesizing evidence on B-cell-targeted therapy from several clinical trials spanning five different autoimmune diseases—PV, RA, MG, SSc and SS—from the studies published between 2020 and 2025. The efficacy of B-cell-targeted therapy in autoimmunity was well established by several landmark trials conducted much before. The RITUX3 trial (2017) in PV demonstrated superior long-term efficacy of RTX, transforming the landscape of pemphigus management (71). Similarly, REFLEX (2006) and DANCER (2006) studies provided a breakthrough for the management of methotrexate- and TNF inhibitor-refractory RA patients, demonstrating significant improvement in the disease activity following RTX treatment (72, 73). However, earlier trials in SS (TEARS-2014 and TRACTISS-2014), provided evidence of limited clinical efficacy using RTX monotherapy, reinforcing the need to develop combinatorial therapies, biosimilars, and next-generation engineered cell therapies (30, 74). Therefore, in this review, our objective is to assess the cotemporary advancements in B-cell therapeutics in autoimmune conditions in the past 5 years including the follow-up study of the landmark RITUX3 trial. The trials in the review encompass variations in B-cell therapeutic strategies, disease mechanism, study design and clinical outcomes, and follow-up durations. Despite substantial heterogeneity, this review highlights several consistent and biologically meaningful patterns underscoring the central role of B cells in autoimmune pathogenesis in current therapeutics.

The involvement of B cells in autoimmunity extends beyond the production of autoantibodies. B cells are critical antigen-presenting cells to T cells and secrete cytokines affecting the functionality of other immune as well as non-immune cells in its microenvironment (11, 36). They are involved in the germinal center as well as the formation of long-lived plasma cells responsible for the disease relapse and short durability of contemporary therapeutics. Furthermore, B-cell centrality is also determined by their interaction with fibroblast cells and stromal cells, resulting in the formation of tertiary lymphoid structures, another factor responsible for the persistence of disease and relapse as well as therapeutic resistance (75). Therefore, success and therapeutic efficacy are dependent not on a single factor but rather on the multifaceted involvement of B cells in disease biology.

### Overall effectiveness of B-cell-targeted therapies

The findings from the current review validate this mechanistic work into clinical efficacy that depends on the disruption of B-cell pathways. PV and MG are autoimmune diseases strongly driven by autoantibody production, thereby reinforcing the relevance of targeting antibody-producing B cells (76–78). Clinical trials in this review demonstrated the most superior and consistent efficacy of B-cell depletion therapy in these autoimmune conditions (46–48, 63). In both, B cells were depleted either with anti-CD20 RTX or with anti-CD19 inebilizumab. RTX consistently exhibited superior clinical response with CR off therapy and durable benefit as followed up to 7 years in PV (46–48). Furthermore, in MG, inebilizumab as well as RTX resulted in significant improvements in MG-ADL scores and functional outcomes accompanied by sustained benefit (63, 65). These findings highlight the fact that the success of B-cell therapy depends on the B-cell centrality to the disease.

However, other diseases with complex immune mechanisms such as RA and SSc documented variable clinical outcomes. Although B-cell therapy including RTX, its biosimilars, BTK inhibition, and anti-CD22 antibody resulted in improvement in disease activity and serum markers in RA, it mainly corresponded to prior treatment and activity scores, as well as the tissue-specific immune architecture (51–57, 59, 60). Furthermore, biopsy-guided trials indicated the importance of B-cell molecular profiling of synovial tissue identifying B-cell-rich/poor signatures along with histological classification to predict treatment outcomes in RA, underscoring the significance of precision medicine in complex diseases (59, 60). SSc also demonstrated skin improvement following RTX, but current evidence remains constrained by short follow-ups and a small study cohort (67, 68). B-cell therapy provides clinical improvement but fails to achieve clinical remission in these complex conditions. Previous literature suggests RA as a multifaceted condition wherein the B cell is a critical player, but there is also a complex interplay of immune cells like T cells, fibroblast-like synoviocytes, and M1 macrophages, and the development of tertiary lymphoid tissues that drive the disease progression (79–83). Apart from this, many cases of RA are seronegative where B-cell depletion is insufficient to provide clinical response (84, 85). Similarly, SSc is driven by macrophages and mast cells in tissues, alongside B cells, and also involves fibroblasts and vascular pathways that may not necessarily be disrupted by B-cell therapeutics (86, 87).

### Dissecting the mechanistic basis for variable efficacy among B-cell therapies

Because variable mechanisms of B-cell therapies including direct depletion of B cells using key markers like CD19/CD20 often bring rapid clinical responses, incomplete depletion of all B-cell subsets, such as long-lived plasma cells in bone marrow that have downregulated CD19 or CD20 surface expression, results in variable response and disease relapse due to repopulation of B cells from the remaining precursor or memory B cells (88, 89). This underscores the need for developing newer targets for therapeutics.

Findings from the other trials suggested alternative strategies to modulate B cells not by depletion but by targeting B-cell signaling. BTK inhibition is one such strategy that works by targeting B-cell receptor signaling and affecting B-cell activation and cytokine secretion (90). BTK inhibition as demonstrated by trials in PV and RA, despite resulting in rapid disease control and reduced steroid burden, had lower CR rates compared to RTX, suggesting the inadequate efficacy of BTK inhibition therapy to produce durable clinical outcomes in diseases dominated by either autoantibodies or complex immune mechanisms (49–51). However, BTK inhibitors demonstrated variable efficacy in RA such that TAS5315 and fenebrutinib improved the disease activity while BMS-986142 did not achieve the primary endpoint even after reducing immunoglobulin levels and plasma cell-associated gene expression (51, 61). Similarly, despite a successful phase 2 BELIEVE trial, the phase 3 PEGASUS trial failed to achieve its primary endpoint of CR using rilzabrutinib and achieving just 24% remission rate compared to 18% in placebo (91), resulting in the discontinuation of rilzabrutinib in the clinical development of PV. This highlights the limitations of BTK inhibition therapy to be used as a stand-alone therapy for durable responses in certain disease conditions like PV.

However, cutting-edge approaches like CAR-T cells can target these long-lived plasma cells as seen in the MG trial (64). Despite the preliminary and small cohort, this trial presented a proof of concept that more comprehensive targeting of B-cell lineage might be required to achieve CR in certain autoimmune conditions. In MG, BCMA-directed CAR-T cell therapy, which targets both long-lived plasma cells, showed promising results of early clinical improvements and decreased antibody titers and BAFF/APRIL levels (64). Additionally, CAR-T cell therapy resulted in prolonged and drug-free remission in multiple autoimmune conditions like SLE and SSc (92).

### Durability of response

While RTX has reported durable and long benefits of up to 7 years in PV, disease variability and patient responsiveness pose a challenge to provide benefit to autoimmune conditions (48). The outcomes from the SS trial bring out an alternative yet effective strategy to modulate B cells via combinatorial therapy (66). The trial concluded that RTX alongside belimumab resulted in durable and profound depletion of B cells accompanied by a reduction in B-cell activation and disease activity compared to monotherapy (66). The findings support the idea that targeting both depletion and B-cell survival and activation pathways, in diseases displaying incomplete responses to monotherapies, might enhance responses through combinatorial or sequential therapies. As previously reported, loss of B cells resulted in BAFF spike in the serum (93). Since BAFF acts as a stimulator for B-cell maturation and activation, it can repopulate B cells via the remaining memory or precursor B cells (94). Therefore, further BAFF blockade can lead to stringent B-cell depletion while increasing clinical efficacy (95).

### Safety considerations

The safety profiles of B-cell therapies across all the reviewed autoimmune diseases were generally well tolerated with mild to moderate adversity. Prolonged follow-up data from PV and RA also suggest no unexpected safety outcomes, indicating the persistent use of B-cell therapy as a pivotal therapeutic strategy in certain autoimmune conditions. However, the use of RTX and leflunomide therapy in RA demonstrated higher incidence of SAEs, highlighting the need for clinical vigilance while combining immunosuppressive agents (62).

### Ongoing clinical trials

Another notable finding of this review is the identification of ongoing trials in autoimmune diseases. There is a rapid evolution of the clinical development landscape of B-cell therapeutics in these conditions. An increased emphasis has been given on the comprehensive and more durable therapeutic responses through simultaneous targeting of long-lived plasma cells and autoreactive B cells. Most ongoing trials are utilizing CAR-T cell platforms to target CD19/CD20/BCMA along with BAFF/APRIL approaches (70). CAR-T platforms provide a novel approach of selective immune reprogramming rather than sustained immunosuppression. In autoimmunity, CAR-T cells support the concept of immune reset such that autoreactive B cells, memory B cells, and long-lived plasma cells are replaced by naïve B-cell repertoire (96, 97). This may contribute towards the restoration of immune tolerance and durable drug-free responses. Although most trials are in the early phase, the use of modern engineered cell therapies and combinatorial therapies may redefine the therapeutic landscape of refractory or B-cell-mediated autoimmune diseases substantially in the coming years.

## Conclusion

**Figure 4:**
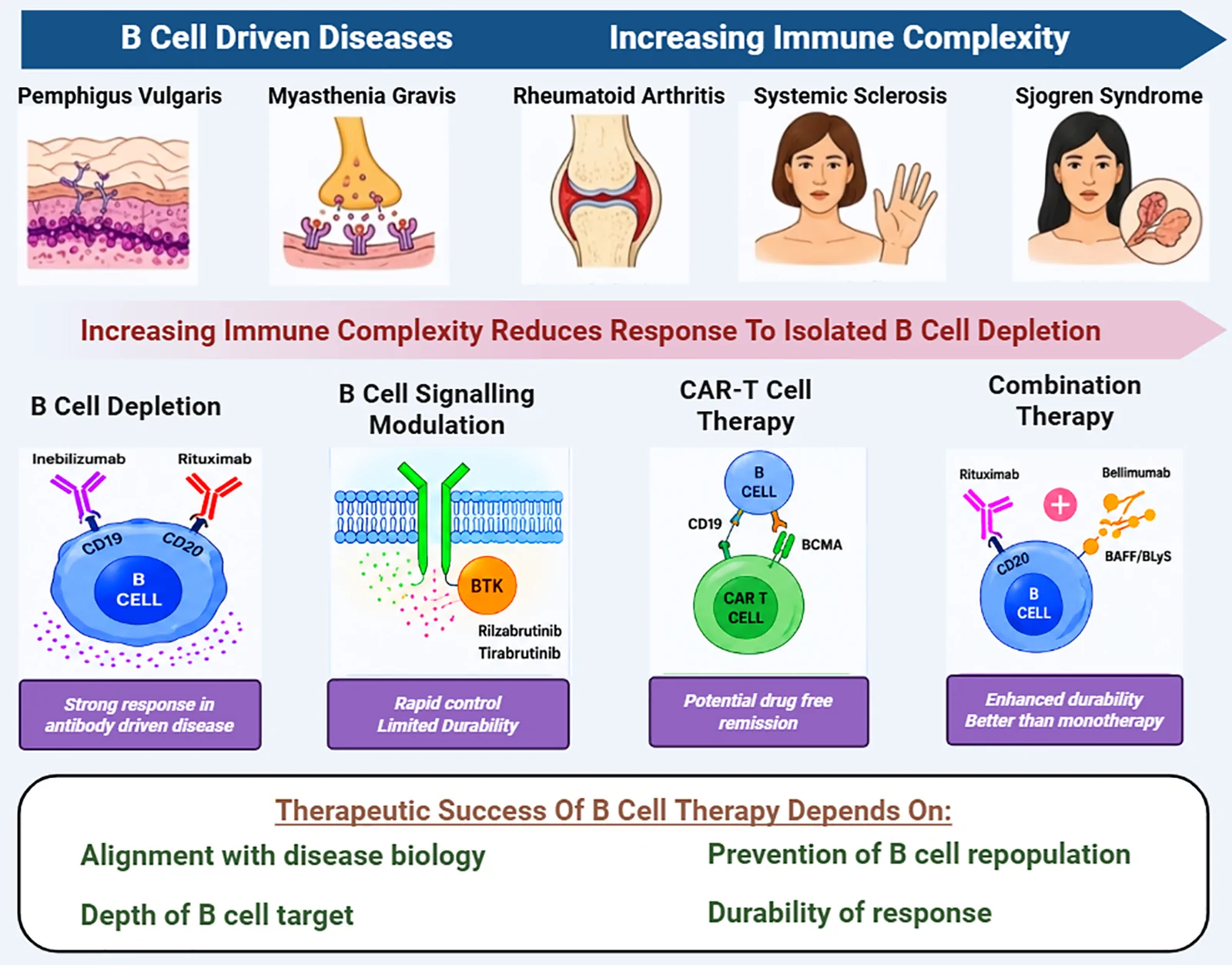
B-cell centrality and implications of B-cell therapeutics in autoimmunity. This image summarizes the systematic review. Increasing immune complexity attributed to reduce response through isolated B-cell depletion (monotherapy). Next-generation CAR-T cell and combination therapy present a better outcome and enhanced durability of clinical responses.

This comprehensive review highlighted the overarching function of B cells as therapeutic target in autoimmune disease. Clinical efficacy appears to be determined by the timely and strategic target of B cells and not just its systemic depletion. Whether a complete reboot via CAR-T therapy or the combinatorial therapy is used, the precision of B-cell therapy on the basis of disease biology decides the therapeutic success (**Figure 4**). However, limitations of this review include the heterogeneity of diseases, trials, study designs, and follow-ups and variable therapeutic strategies that constrained direct quantitative comparison or meta-analysis. The findings should be considered within the disease immunopathology context rather than a universal representation of autoimmune disease pathogenesis. Nonetheless, decades of mechanistic and functional B-cell studies translated into these clinical trials strengthen the robustness of B cells as one of the key mediators of autoimmune diseases.

### Unmet needs and future perspectives

Future therapeutics should extend beyond conventional B-cell depletion strategies to combinatorial as well as next-generation engineered cellular therapies to target deeper issues of disease relapse and persistence. BCMA directed along with the B-cell depletion (CD19/20) approach may improve clinical outcomes by targeting more comprehensive eradication of autoreactive B-cell lineage consisting of long-lived plasma cells pertaining to relapse. Additionally, the use of BAFF/APRIL inhibitors following depletion may ensure the durability of responses by addressing compensatory mechanisms causing the repopulation of autoreactive B cells. The clinical trials to be conducted in the future should also prioritize longer follow-up to access durability of responses, the identification of early biomarkers to distinguish responders from non-responders, the identification of alternative strategies for low- or middle-income countries such as low-dose trials, and the development of standardized definitions of CR or pathological response for each autoimmune condition. Additionally, the integration of molecular tissue profiling in clinical settings will help validate transcriptomic and cellular signatures and facilitate the development of precision medicine. Comparative approaches to monitor B-cell depletion therapy and B-cell modulation therapy, such as signaling-based approaches, are required for a better understanding of B-cell pathology.

## Data Availability

The original contributions presented in the study are included in the article/**Supplementary Material**. Further inquiries can be directed to the corresponding author.

## Glossary

ACPA: Anti-citrullinated protein antibody
ACR: American College of Rheumatology
ADA: Anti-drug antibody
AE: Adverse event
AECG: American–European Consensus Group
APRIL: A proliferation-inducing ligand
AUCinf: Area under the concentration–time curve to infinity
AZA: Azathioprine
BAFF: B-cell activating factor (B lymphocyte stimulator
BCMA: B-cell maturation antigen
BTK: Bruton’s tyrosine kinase
CAR-T: Chimeric antigen receptor T cell
CDAI: Clinical Disease Activity Index
ClinESSDAI: Clinical European League Against Rheumatism Sjögren’s Syndrome Disease Activity Index
CR: Complete remission
CRP: C-reactive protein
DAS: Disease Activity Score
DFS: Disease-free survival
DLQI: Dermatology Life Quality Index
DLTs: Dose-limiting toxicities
DMARDs: Disease-modifying anti-rheumatic drugs
DSG: Desmoglein
ESSDAI: European League Against Rheumatism Sjögren’s Syndrome Disease Activity Index
EULAR: European Alliance of Associations for Rheumatology
FVC: Forced vital capacity
HAQ-DI: Health Assessment Questionnaire–Disability Index
HBV: Hepatitis B virus
HCV: Hepatitis C virus
HIV: Human immunodeficiency virus
HTN: Hypertension
IR: Infusion reaction
IV: Intravenous
IVIG: Intravenous immunoglobulin
JAK: Janus kinase
LDA: Low disease activity
mAb: Monoclonal antibody
MG: Myasthenia gravis
MG-ADL: Myasthenia Gravis–Activities of Daily Living
MGFA: Myasthenia Gravis Foundation of America
MMF: Mycophenolate mofetil
mRSS: Modified Rodnan Skin Score
MTX: Methotrexate
MuSK: Muscle-specific kinase
NAb: Neutralizing antibody
NR: Not reported
NSAID: Non-steroidal anti-inflammatory drug
PDAI: Pemphigus Disease Area Index
PF: Pemphigus foliaceus
PK/PD: Pharmacokinetics/pharmacodynamics
PLEX: Plasma exchange
PROs: Patient-reported outcomes
PV: Pemphigus vulgaris
QMG: Quantitative Myasthenia Gravis Score
QoL: Quality of life
RA: Rheumatoid arthritis
RAID: Rheumatoid arthritis impact of disease
RF: Rheumatoid factor
SAEs: Serious adverse events
SDAI: Simplified Disease Activity Index
SP-D: Surfactant protein D
SSc: Systemic sclerosis
SSA/B: Sjögren’s syndrome–related antigen A/B
SS: Sjögren’s syndrome
TB: Tuberculosis
TEAE: Treatment-emergent adverse event
Tfh: T follicular helper cell.
