# Supplementary material for: B-cell centrality dictates therapeutic efficacy across autoimmune diseases: a systematic review
Source: Front Immunol. 2026 Sep 10;17:1906254. doi: 10.3389/fimmu.2026.1906254 (PMC13600982; doi:10.3389/fimmu.2026.1906254)
Supplement: Supplementary file 1 [file Table1.docx]

**Supplementary file**

**PRISMA abstract checklist**

| **Section and Topic** | **Item #** | **Checklist item** | **Reported (Yes/No)** |
| --- | --- | --- | --- |
| **TITLE** | | |  |
| Title | 1 | Identify the report as a systematic review. | yes |
| **BACKGROUND** | | |  |
| Objectives | 2 | Provide an explicit statement of the main objective(s) or question(s) the review addresses. | yes |
| **METHODS** | | |  |
| Eligibility criteria | 3 | Specify the inclusion and exclusion criteria for the review. | yes |
| Information sources | 4 | Specify the information sources (e.g. databases, registers) used to identify studies and the date when each was last searched. | yes |
| Risk of bias | 5 | Specify the methods used to assess risk of bias in the included studies. | no |
| Synthesis of results | 6 | Specify the methods used to present and synthesise results. | yes |
| **RESULTS** | | |  |
| Included studies | 7 | Give the total number of included studies and participants and summarise relevant characteristics of studies. | yes |
| Synthesis of results | 8 | Present results for main outcomes, preferably indicating the number of included studies and participants for each. If meta-analysis was done, report the summary estimate and confidence/credible interval. If comparing groups, indicate the direction of the effect (i.e. which group is favoured). | yes |
| **DISCUSSION** | | |  |
| Limitations of evidence | 9 | Provide a brief summary of the limitations of the evidence included in the review (e.g. study risk of bias, inconsistency and imprecision). | no |
| Interpretation | 10 | Provide a general interpretation of the results and important implications. | yes |
| **OTHER** | | |  |
| Funding | 11 | Specify the primary source of funding for the review. | no |
| Registration | 12 | Provide the register name and registration number. | no |

**PRISMA 2020 Checklist**

| **Section and Topic** | **Item #** | **Checklist item** | **Location where item is reported** |
| --- | --- | --- | --- |
| **TITLE** | | |  |
| Title | 1 | Identify the report as a systematic review. | Page 1 |
| **ABSTRACT** | | |  |
| Abstract | 2 | See the PRISMA 2020 for Abstracts checklist. | Abstract |
| **INTRODUCTION** | | |  |
| Rationale | 3 | Describe the rationale for the review in the context of existing knowledge. | introduction |
| Objectives | 4 | Provide an explicit statement of the objective(s) or question(s) the review addresses. | introduction |
| **METHODS** | | |  |
| Eligibility criteria | 5 | Specify the inclusion and exclusion criteria for the review and how studies were grouped for the syntheses. | Methods: inclusion and exclusion criteria |
| Information sources | 6 | Specify all databases, registers, websites, organisations, reference lists and other sources searched or consulted to identify studies. Specify the date when each source was last searched or consulted. | Methods: data sources and search strategy |
| Search strategy | 7 | Present the full search strategies for all databases, registers and websites, including any filters and limits used. | Methods: data sources and search strategy and supplementary table 1 |
| Selection process | 8 | Specify the methods used to decide whether a study met the inclusion criteria of the review, including how many reviewers screened each record and each report retrieved, whether they worked independently, and if applicable, details of automation tools used in the process. | Methods: data sources and search strategy, inclusion and exclusion criteria |
| Data collection process | 9 | Specify the methods used to collect data from reports, including how many reviewers collected data from each report, whether they worked independently, any processes for obtaining or confirming data from study investigators, and if applicable, details of automation tools used in the process. | Methods: data sources and search strategy, inclusion and exclusion criteria |
| Data items | 10a | List and define all outcomes for which data were sought. Specify whether all results that were compatible with each outcome domain in each study were sought (e.g. for all measures, time points, analyses), and if not, the methods used to decide which results to collect. | Methods: data sources and search strategy, inclusion and exclusion criteria |
|  | 10b | List and define all other variables for which data were sought (e.g. participant and intervention characteristics, funding sources). Describe any assumptions made about any missing or unclear information. | Methods: data sources and search strategy, inclusion and exclusion criteria |
| Study risk of bias assessment | 11 | Specify the methods used to assess risk of bias in the included studies, including details of the tool(s) used, how many reviewers assessed each study and whether they worked independently, and if applicable, details of automation tools used in the process. | Methods: Risk of Bias Assessment |
| Effect measures | 12 | Specify for each outcome the effect measure(s) (e.g. risk ratio, mean difference) used in the synthesis or presentation of results. | Not applicable (qualitative systematic review; no meta-analysis performed) |
| Synthesis methods | 13a | Describe the processes used to decide which studies were eligible for each synthesis (e.g. tabulating the study intervention characteristics and comparing against the planned groups for each synthesis (item #5)). | Methods: Inclusion/Exclusion Criteria & Data Screening |
|  | 13b | Describe any methods required to prepare the data for presentation or synthesis, such as handling of missing summary statistics, or data conversions. | Methods: data sources and search strategy, inclusion and exclusion criteria |
|  | 13c | Describe any methods used to tabulate or visually display results of individual studies and syntheses. | Results: Table 2-5 |
|  | 13d | Describe any methods used to synthesize results and provide a rationale for the choice(s). If meta-analysis was performed, describe the model(s), method(s) to identify the presence and extent of statistical heterogeneity, and software package(s) used. | Not applicable |
|  | 13e | Describe any methods used to explore possible causes of heterogeneity among study results (e.g. subgroup analysis, meta-regression). | Discussion |
|  | 13f | Describe any sensitivity analyses conducted to assess robustness of the synthesized results. | Not applicable |
| Reporting bias assessment | 14 | Describe any methods used to assess risk of bias due to missing results in a synthesis (arising from reporting biases). | Not performed |
| Certainty assessment | 15 | Describe any methods used to assess certainty (or confidence) in the body of evidence for an outcome. | Not performed |
| **RESULTS** | | |  |
| Study selection | 16a | Describe the results of the search and selection process, from the number of records identified in the search to the number of studies included in the review, ideally using a flow diagram. | Results and Figure 2 |
|  | 16b | Cite studies that might appear to meet the inclusion criteria, but which were excluded, and explain why they were excluded. | Figure 2 |
| Study characteristics | 17 | Cite each included study and present its characteristics. | Results and table 2 and 3 |
| Risk of bias in studies | 18 | Present assessments of risk of bias for each included study. | Results and Figure 3 |
| Results of individual studies | 19 | For all outcomes, present, for each study: (a) summary statistics for each group (where appropriate) and (b) an effect estimate and its precision (e.g. confidence/credible interval), ideally using structured tables or plots. | Table 4 |
| Results of syntheses | 20a | For each synthesis, briefly summarise the characteristics and risk of bias among contributing studies. | Results |
|  | 20b | Present results of all statistical syntheses conducted. If meta-analysis was done, present for each the summary estimate and its precision (e.g. confidence/credible interval) and measures of statistical heterogeneity. If comparing groups, describe the direction of the effect. | Not applicable |
|  | 20c | Present results of all investigations of possible causes of heterogeneity among study results. | Not applicable/ discussion |
|  | 20d | Present results of all sensitivity analyses conducted to assess the robustness of the synthesized results. | Not applicable |
| Reporting biases | 21 | Present assessments of risk of bias due to missing results (arising from reporting biases) for each synthesis assessed. | Results |
| Certainty of evidence | 22 | Present assessments of certainty (or confidence) in the body of evidence for each outcome assessed. | Not applicable |
| **DISCUSSION** | | |  |
| Discussion | 23a | Provide a general interpretation of the results in the context of other evidence. | Discussion |
|  | 23b | Discuss any limitations of the evidence included in the review. | Discussion |
|  | 23c | Discuss any limitations of the review processes used. | Discussion |
|  | 23d | Discuss implications of the results for practice, policy, and future research. | Conclusion and Unmet and future prospectives |
| **OTHER INFORMATION** | | |  |
| Registration and protocol | 24a | Provide registration information for the review, including register name and registration number, or state that the review was not registered. | Not registered |
|  | 24b | Indicate where the review protocol can be accessed, or state that a protocol was not prepared. | Not prepared |
|  | 24c | Describe and explain any amendments to information provided at registration or in the protocol. | Not applicable |
| Support | 25 | Describe sources of financial or non-financial support for the review, and the role of the funders or sponsors in the review. | Funding information |
| Competing interests | 26 | Declare any competing interests of review authors. | Conflict of interest |
| Availability of data, code and other materials | 27 | Report which of the following are publicly available and where they can be found: template data collection forms; data extracted from included studies; data used for all analyses; analytic code; any other materials used in the review. | Not applicable |

**Table S1:** A tabular representation illustrating the search strategies employed by different search engines.

| **Database source** | **Search string** | **Results** |
| --- | --- | --- |
| **Embase** | (('b cell targeted therapies' OR (b AND ('cell'/exp OR cell) AND targeted AND therapies) OR 'b cell therapies' OR (b AND ('cell'/exp OR cell) AND therapies) OR 'anti cd20' OR (anti AND cd20) OR 'anti cd19' OR (anti AND cd19) OR 'rituximab'/exp OR rituximab OR 'ocrelizumab'/exp OR ocrelizumab OR 'ofatumumab'/exp OR ofatumumab OR 'ublituximab'/exp OR ublituximab OR 'inebilizumab'/exp OR inebilizumab OR 'b cell trials' OR (b AND ('cell'/exp OR cell) AND trials) OR 'belimumab'/exp OR belimumab OR 'cart b cell' OR (cart AND b AND ('cell'/exp OR cell))) AND ('pemphigus vulgaris'/exp OR 'pemphigus vulgaris' OR (('pemphigus'/exp OR pemphigus) AND vulgaris)) OR 'rheumatoid arthritis'/exp OR 'rheumatoid arthritis' OR (rheumatoid AND ('arthritis'/exp OR arthritis)) OR 'sjoegren syndrome'/exp OR 'sjoegren syndrome' OR (sjoegren AND ('syndrome'/exp OR syndrome)) OR 'systemic sclerosis'/exp OR 'systemic sclerosis' OR (systemic AND ('sclerosis'/exp OR sclerosis)) OR 'myasthenia gravis'/exp OR 'myasthenia gravis' OR (('myasthenia'/exp OR myasthenia) AND gravis)) AND ([controlled clinical trial]/lim OR [randomized controlled trial]/lim) AND [2020-2025]/py AND [01-01-2020]/sd NOT [01-07-2025]/sd AND [embase]/lim NOT ([embase]/lim AND [medline]/lim) AND ('myasthenia gravis'/dm OR 'rheumatoid arthritis'/dm OR 'sjoegren syndrome'/dm OR 'systemic sclerosis'/dm) AND ('clinical trial'/de OR 'controlled study'/de OR 'human'/de OR 'randomized controlled trial'/de) AND 'article'/it | 411 |
| **Web of Science** | b cell targeted therapies OR b cell therapies OR anti cd20 OR anti cd19 OR rituximab OR ocrelizumab OR ofatumumab OR ublituximab OR inebilizumab OR b cell trials OR belimumab OR cart b cell (All Fields) and pemphigus vulgaris OR rheumatoid arthritis OR sjoegren syndrome OR systemic sclerosis OR myasthenia gravis (Topic) and Trial OR clinical trial OR randomized trial (All Fields) not non-human studies OR animal studies OR conference abstracts OR case reports OR observational studies OR meta-analysis OR reviews OR other autoimmune diseases (All Fields) and Article (Document Types) | 313 |
| **PubMed** | (((((((((((((b cell targeted therapies) OR (b cell therapies)) OR (anti cd20)) OR (anti cd19)) OR (rituximab)) OR (ocrelizumab)) OR (ofatumumab)) OR (ublituximab)) OR (inebilizumab)) OR (b cell trials)) OR (belimumab)) OR (cart b cell)) ) AND (pemphigus vulgaris) Filters: Clinical Study, Clinical Trial, Clinical Trial, Phase I, Clinical Trial, Phase II, from 2020 – 2025  ((((((((((((((b cell targeted therapies) OR (b cell therapies)) OR (anti cd20)) OR (anti cd19)) OR (rituximab)) OR (ocrelizumab)) OR (ofatumumab)) OR (ublituximab)) OR (inebilizumab)) OR (b cell trials)) OR (belimumab)) OR (cart b cell)) ) ) AND (rheumatoid arthritis) Filters: Clinical Study, Clinical Trial, Clinical Trial, Phase I, Clinical Trial, Phase II, Randomized Controlled Trial, from 2020 – 2025   (((((((((((((((b cell targeted therapies) OR (b cell therapies)) OR (anti cd20)) OR (anti cd19)) OR (rituximab)) OR (ocrelizumab)) OR (ofatumumab)) OR (ublituximab)) OR (inebilizumab)) OR (b cell trials)) OR (belimumab)) OR (cart b cell)) ) ) ) AND (myasthenia gravis) Filters: Clinical Study, Clinical Trial, Clinical Trial, Phase I, Clinical Trial, Phase II, Randomized Controlled Trial, from 2020 – 2025  ( (((((((((((((((b cell targeted therapies) OR (b cell therapies)) OR (anti cd20)) OR (anti cd19)) OR (rituximab)) OR (ocrelizumab)) OR (ofatumumab)) OR (ublituximab)) OR (inebilizumab)) OR (b cell trials)) OR (belimumab)) OR (cart b cell)) ) ) ) ) AND (systemic sclerosis) Filters: Clinical Study, Clinical Trial, Clinical Trial, Phase I, Clinical Trial, Phase II, Randomized Controlled Trial, from 2020 – 2025  (( (((((((((((((((b cell targeted therapies) OR (b cell therapies)) OR (anti cd20)) OR (anti cd19)) OR (rituximab)) OR (ocrelizumab)) OR (ofatumumab)) OR (ublituximab)) OR (inebilizumab)) OR (b cell trials)) OR (belimumab)) OR (cart b cell)) ) ) ) ) ) AND (Sjogren syndrome) Filters: Clinical Study, Clinical Trial, Clinical Trial, Phase I, Clinical Trial, Phase II, Randomized Controlled Trial, from 2020 - 2025 | 255 |
